# Bone marrow-derived mesenchymal stem cells enhance autophagy *via* PI3K/AKT signalling to reduce the severity of ischaemia/reperfusion-induced lung injury

**DOI:** 10.1111/jcmm.12638

**Published:** 2015-07-14

**Authors:** Jing Li, Jian Zhou, Dan Zhang, Yuanlin Song, Jun She, Chunxue Bai

**Affiliations:** a Department of Pulmonary Medicine, Zhongshan Hospital, Fudan University, Shanghai Respiratory Research InstituteShanghai, China

**Keywords:** autophagy, bone marrow-derived mesenchymal stem cells, lung injury

## Abstract

Autophagy, a type II programmed cell death, is essential for cell survival under stress, *e.g*. lung injury, and bone marrow-derived mesenchymal stem cells (BM-MSCs) have great potential for cell therapy. However, the mechanisms underlying the BM-MSC activation of autophagy to provide a therapeutic effect in ischaemia/reperfusion-induced lung injury (IRI) remain unclear. Thus, we investigate the activation of autophagy in IRI following transplantation with BM-MSCs. Seventy mice were pre-treated with BM-MSCs before they underwent lung IRI surgery *in vivo*. Human pulmonary micro-vascular endothelial cells (HPMVECs) were pre-conditioned with BM-MSCs by oxygen-glucose deprivation/reoxygenation (OGD) *in vitro*. Expression markers for autophagy and the phosphoinositide 3-kinase/protein kinase B (PI3K/Akt) signalling pathway were analysed. In IRI-treated mice, administration of BM-MSCs significantly attenuated lung injury and inflammation, and increased the level of autophagy. In OGD-treated HPMVECs, co-culture with BM-MSCs attenuated endothelial permeability by decreasing the level of cell death and enhanced autophagic activation. Moreover, administration of BM-MSCs decreased the level of PI3K class I and p-Akt while the expression of PI3K class III was increased. Finally, BM-MSCs-induced autophagic activity was prevented using the inhibitor LY294002. Administration of BM-MSCs attenuated lung injury by improving the autophagy level *via* the PI3K/Akt signalling pathway. These findings provide further understanding of the mechanisms related to BM-MSCs and will help to develop new cell-based therapeutic strategies in lung injury.

## Introduction

Regenerative medicine using human stem cells is a novel strategy for the treatment of various intractable diseases and damaged organs, including difficult-to-treat lung diseases 1. Many studies have demonstrated that bone marrow-derived mesenchymal stem cells (BM-MSCs) appear to exert anti-inflammation and anti-apoptosis effects and represent a promising field for the treatment of acute lung injury (ALI) and acute respiratory distress syndrome 2,3. However, their function and underlying mechanism in ischaemia/reperfusion-induced lung injury (IRI) remain largely unknown.

Macroautophagy (referred hereafter as autophagy) is a type of programmed cell death that is different from apoptosis and necrosis and is a degradation process by which cytoplasmic material is sequestered in a double-membrane vesicle destined for degradation 4. Although autophagy is sometimes associated with cell death, it is generally considered to be a survival mechanism because autophagy protects cells against cellular distress, especially hypoxia, starvation and infection 5,6. Thus, it has been argued that autophagy is a crucial process that regulates cell survival, development and homoeostasis. Recently, the implications of autophagy in lung disease have been demonstrated, such as pulmonary hypertension and chronic obstructive pulmonary disease 7,8. To the best of our knowledge, an autophagic mechanism has not yet been evaluated in BM-MSCs-related IRI lung injury therapy.

The autophagy signal is complicated, and there are several pathways that are linked to autophagy signalling 9. The phosphoinositide 3-kinase/protein kinase B (PI3K/Akt) pathway can be activated by a variety of extracellular stimuli and regulate a wide range of cellular processes 10. Furthermore, it has emerged as a key regulator of autophagy because of its role in cell survival and proliferation 11,12. Numerous studies have examined this pathway, but whether it participates in the effects of BM-MSCs on IRI remains unclear.

Thus, the aims of this study were to investigate whether BM-MSCs transplantation influences the autophagic mechanism to attenuate damage of the injured lung and to determine whether the PI3K/Akt signalling pathway is involved in this process.

## Materials and methods

### Cell culture

Human BM-MSCs and human pulmonary micro-vascular endothelial cells (HPMVECs) were purchased from ScienCell (Carlsbad, CA, USA). Vials of 0.5 × 10^6^ cells (passage 1) were thawed, plated onto culture chambers (Corning, Lowell, MA, USA) in mesenchymal stem cell medium and endothelial cell growth medium (ECM) (ScienCell), and incubated at 37°C in 5% CO_2_ for 6–7 days until 85–90% confluency was reached. The cells were used at passages 3–5. Normal adult human lung fibroblasts (ScienCell), used as control cells, were cultured in DMEM (Invitrogen, Carlsbad, CA, USA) supplemented with 10% fetal bovine serum (FBS) (Invitrogen) and 1% penicillin/streptomycin.

### Mice model of lung injury

A total of 70 male C57BL/6J mice (8–10 weeks) were purchased from Shanghai Laboratory Animal Center (China). All of the study protocols were approved by the Animal Care Committees of Fudan University. All animals were cared for according to the Guide for the Care and Use of Laboratory Animals published by the National Academy of Sciences 13.

Animals were randomly grouped into four groups (*n* = 15 for each group): (*i*) animals pre-treated with PBS and undergoing a sham operation (Control); (*ii*) animals undergoing a lung IRI operation (IRI); (*iii*) animals pre-treated with BM-MSCs and undergoing a sham operation (Control+ MSC); and (*iv*) animals pre-treated with BM-MSCs and undergoing a lung IRI operation (IRI+MSC). Left lung IRI operations were performed as previously described 14,15. A total of 5 × 10^5^ BM-MSCs suspended in 100 μl of PBS or the same volume of PBS without BM-MSCs was injected *via* the tail vein 1 hr before surgery. Next, mice were anaesthetized *via* intraperitoneal administration of 60 mg/kg pentobarbital sodium salt. After cannulation, the mice were mechanically ventilated (Harvard Apparatus ASV, 55-7059, Holliston, MA, USA) at a 0.28 ml tidal volume and 80/min. respiratory rate. The mice were placed on their right sides, and a left anterolateral thoracotomy in the fifth intercostal space was performed. The left hilum was clamped for 30 min. After 30 min. of left lung ischaemia, the clamp was removed and the lung was ventilated and reperfused for 120 min. At the end of the reperfusion period, lung tissues were harvested for further analysis.

### Histological examination

The fresh left lung was fixed in a 10% formaldehyde solution for 24 hrs and then embedded in paraffin. Four-micrometre thick sections were stained with haematoxylin and eosin and then examined using light microscopy (Nikon eclipse Ti-U, Tokyo, Japan) by a pathologist who was blind to the experimental groups. Lung injury was assessed according to a 0–4 point scale as previously described 16: 0 = normal; 1 = minimal (<25%); 2 = mild (25–50%); 3 = moderate (50–75%); and 4 = severe (>75%).

### Lung wet-to-dry ratio

The left lung was harvested at the end of the surgical procedure. The trachea and oesophagus were separated from the lung, and the lung was weighed immediately. Subsequently, the lung was stored at 56°C to dry for 72 hrs and reweighed to calculate the wet-to-dry ratio.

### ELISA

After left lung IRI operations, the mice were killed and bronchoalveolar lavage was performed through a tracheal cannula with 0.8 ml of PBS four times to obtain the bronchoalveolar lavage fluid (BALF). Approximately 2.5 ml of BALF was obtained from each mouse. The recovered lavage fluid was then centrifuged at 800 × g for 10 min. at 4°C and the supernatants were stored at −80°C until further use. The BALF levels of tumour necrosis factor-α (TNF-α) and interleukin-1β (IL-1β) were measured by ELISA according to the manufacturer’s instructions (R&D, Minneapolis, MN, USA). All measurements were performed in duplicate.

### *In vitro* co-culture experiments

To confirm whether BM-MSCs protect HPMVECs under the OGD condition, a Transwell co-culture system was used. HPMVECs were grown in 24-well or 6-well plates coated with rat collagen I (catalogue 08115; Millipore, Billerica, MA, USA) diluted in PBS. The 3rd–5th passages of BM-MSCs or fibroblasts (3 × 10^4^ cells/ml) were seeded in the upper or lower well of the 6-well or 24-well Transwell system and cultured for 2 days, respectively. HPMVECs were randomly divided into: (*i*) Control group; (*ii*) BM-MSCs pre-condition group (Control+ MSC); (*iii*) Oxygen-glucose deprivation/reoxygenation group (OGD); (*iv*) BM-MSCs pre-condition and OGD (OGD+MSC) group; and (*v*) Fibroblasts pre-condition and OGD (OGD+FIB) group. The ECM was replaced with glucose-free DMEM (11966-025; Invitrogen), and the cells were placed in an anaerobic chamber (5% CO_2_, 95% N_2_; Memmert; Schwabach, Germany) for OGD. After 8 hrs, the medium was replaced with ECM and the plates were grown in a normoxic chamber (37°C, 5% CO_2_) for 12 hrs of reoxygenation.

### Endothelial cell permeability

Flux of fluorescein isothiocyanate dextran (FITC-dextran) (MW 40,000; Sigma-Aldrich, St. Louis, MO, USA) across the HPMVECs monolayer was used to measure endothelial cell permeability. Briefly, HPMVECs were seeded in Transwell-Clear chambers (0.4 μm pore size; Costar, Corning, NY, USA) at a cell density of 1 × 10^5^ cells/well. The cells grown in the chambers were visible under a microscope and were examined for confluence prior to the start of the experiments. FITC-dextran (1 mg/ml) was added to the upper chamber of the transwell inserts, and samples were obtained from the lower chamber after a 30-min incubation. The amount of FITC-dextran was determined at an excitation wavelength of 485 nm and an emission wavelength of 510 nm. The permeability was determined by analysing the fluorescein apparent permeability coefficients (P_app_). P_app_ was calculated using the following formula 17:

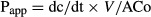
 where dc/dt is the permeability rate (mg/sec.), which is the slope of the plot of the cumulative receiver concentration with time; *V* is the volume of the receiver compartment (ml); A is the membrane surface area (cm^2^); and Co is the initial donor concentration of fluorescein (mg/ml). These results are presented as the percentage of the fluorescence in controls.

### Mitochondrial transmembrane potential (ΔΨm)

ΔΨm was assessed using the lipophilic cationic probe 5,5′,6,6′-Tetrachloro-1,1′,3,3′-tetraethyl-imidacarbocyanine iodide (JC-1 staining, Beyotime Institute of Biotechnology, Jiangsu, China). HPMVECs were incubated with a JC-1 working solution at 37°C in the dark for 20 min. and observed using fluorescence microscopy. In normal cells, where the mitochondrial potentials remain depolarized, JC-1 forms complexes showing punctate red fluorescence at a 590 nm emission wavelength; however, in apoptotic cells, JC-1 remains in its monomeric form, showing diffused green fluorescence at a 530 nm emission wavelength.

### Cell apoptosis and necrosis assay

Human pulmonary micro-vascular endothelial cells or BM-MSCs (1 × 10^6^ cells/ml) were seeded into six-well plates and were subjected to various treatments as previously described. Detection of apoptosis by flow cytometry (FCM) was performed with an Annexin V-FTIC/Propidium iodide (PI) apoptosis detection kit (Sigma-Aldrich). The staining was performed according to the manufacturer’s instructions. Briefly, cells were washed twice with PBS and resuspended in binding buffer. Next, 125 μl of the solution was transferred into 5-ml culture tubes followed by the addition of 5 μl of Annexin V-FITC and PI. After incubated for 15 min. at room temperature in the dark, the apoptotic rates were analysed using FCM (Accuri C6; BD Biosciences, MI, USA).

### Transmission electron microscopy analysis of autophagy ultrastructures

After OGD treatment, HPMVECs were prefixed with 2.5% glutaraldehyde at 4°C overnight, post-fixed in 1% buffered osmium tetroxide, dehydrated in gradient ethanol, embedded in Epon 812, sectioned using a ultramicrotome and stained with uranyl acetate and lead citrate. Sections were observed under a transmission electron microscopy (TEM; CM120; Philips, Eindhoven, the Netherlands). A total of 40 electron microscopic sections were prepared and the autophagy structures of each group were examined in 200 cells.

### Monodansylcadaverine staining

After OGD treatment, HPMVECs were incubated with 0.05 mM monodansylcadaverine (MDC; Sigma-Aldrich) in PBS at 37°C for 60 min. and then fixed with 4% paraformaldehyde for 15 min. After three washes with PBS, patterns of punctate green fluorescence were detected using confocal microscopy (Nikon A1 R) at an excitation wavelength of 492 nm and a detecting emission of 520 nm.

### Western blotting analysis

Proteins were extracted from the left lung and HPMVECs using Radio-Immunoprecipitation Assay (RIPA) buffer (Beyotime Institute of Biotechnology) lysis buffer. The samples were centrifuged at 13,000 × g for 10 min. at 4°C, and the supernatants were collected. Proteins were quantified using a Bicinchoninic Acid (BCA) Protein Assay Kit (Beyotime Institute of Biotechnology) and 40 μg of proteins were separated using 8–10% SDS-PAGE. After electrophoresis, proteins were transferred onto polyvinylidene fluoride membranes (Millipore). The membranes were blocked with blocking solution (Beyotime Institute of Biotechnology) for 1 hr at room temperature. Next, the membranes were incubated using the following primary antibodies overnight at 4°C: rabbit anti-light chain 3 (LC3; 1:1000; Novus Biologicals, Littleton, CO, USA), rabbit anti-SQSTM1/p62 (1:1000; Abcam, Cambridge, MA, USA), rabbit anti-PI3K class I (1:1000; Cell Signaling Technology, Danvers, MA, USA), rabbit anti-PI3K class III (1:1000; Cell Signaling Technology), rabbit anti-pAKT (1:1000; Cell Signaling Technology), rabbit anti-AKT (1:1000; Cell Signaling Technology) and rabbit anti-β–actin (1:1000; Abcam). After three washes, the membranes were incubated with horseradish peroxidase-conjugated secondary antibodies (Beyotime Institute of Biotechnology) for 1 hr at room temperature. The blots were then visualized using enhanced chemiluminescent reagent (ECL) (Beyotime Institute of Biotechnology). The band densities were analysed using Image Quant (FluorChem FC3; ProteinSimple, Santa Clara, CA, USA).

### Statistical analysis

The values of all of the measurements are presented as the mean ± SD. Comparisons between multiple groups were performed with one-way anova with Bonferroni correlation. The analysis was performed with Graphpad Prism 5. Values of *P* < 0.05 were considered statistically significant.

## Results

### BM-MSCs transplantation attenuated damage in IRI lung injury

After IRI surgery, frozen left lung sections were detected using a fluorescence microscope. Engraftment BM-MSCs, which were originally stained with PKH26 (red) before transplantation, were observed in both the control and IRI groups (Fig. S1).

**Figure 1:**
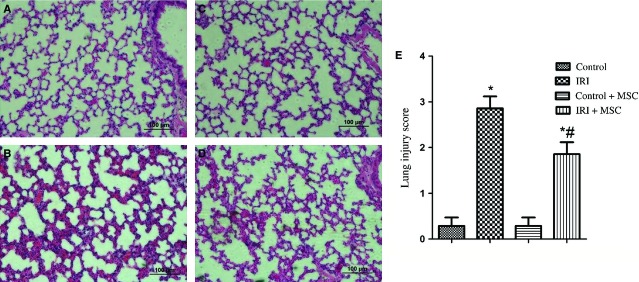
Lung histology using haematoxylin and eosin staining and the lung injury score (*n* = 6 for each group). (A) Control group; (B) IRI group; (C) Control+BM-MSCs group; (D) IRI+ BM-MSCs group; (E) Microscopic injury of the left lung was statistically scored. Data are expressed as the mean ± SD. *P < 0.05 *versus* control group; ^#^*P* < 0.05 *versus* IRI group by anova (Bonferroni).

**Figure 2:**
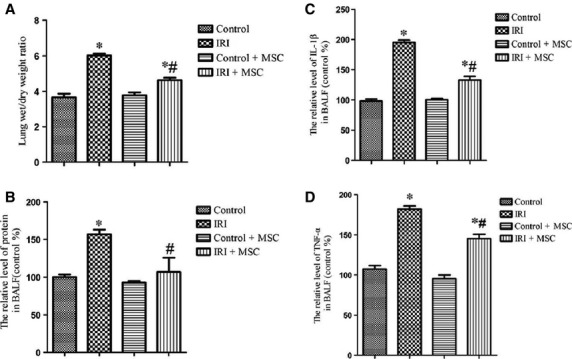
Effects of BM-MSCs on permeability and inflammation in IRI-challenged lungs. Administration of BM-MSCs reduced the level of lung oedema and inflammation compared to the IRI group (*n* = 6 for each group). (A) Treatment with BM-MSCs reduced the wet/dry ratio compared to the IRI group. (B) Pre-conditioning with BM-MSCs attenuated the level of protein in the BALF compared to the IRI group. (C) BM-MSCs decreased the level of IL-1β in the BALF fluid of IRI mice. (D) BM-MSCs decreased the level of TNF-α in the BALF fluid of IRI mice. Data are expressed as the mean ± SD. *P < 0.05 *versus* control group; ^#^*P* < 0.05 *versus* IRI group by anova (Bonferroni).

Ischaemia/reperfusion-induced lung injury significantly damaged lung tissues, with inflammatory cells infiltrating into the lung interstitium and alveolar spaces, alveolar wall thickening, and haemorrhage (Fig. 1A–D). In addition, the lung injury score was highest in the IRI group. However, these histological changes and lung injury score were alleviated in the BM-MSCs pre-treatment group (Fig. 1E). Furthermore, BM-MSCs decreased the lung wet/dry ratio (Fig. 2A) and alveolar fluid protein concentrations (Fig. 2B) compared to the IRI group. One potential mechanism is that BM-MSCs could enhance the restoration of lung micro-vascular permeability.

### BM-MSCs transplantation has an anti-inflammatory effect in IRI lung injury

As shown in Figure 2, the BALF concentrations of IL-1β (Fig. 2C) and TNF-α (Fig. 2D) were decreased following BM-MSCs pre-treatment compared to the IRI group (*P* < 0.05). These results suggest that the transplantation of BM-MSCs has an anti-inflammation effect in injured lung tissues.

### BM-MSCs transplantation activates the autophagic pathway in IRI lung injury

**Figure 3:**
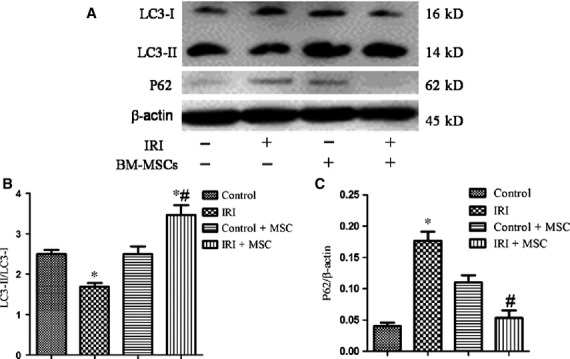
Pre-treatment with BM-MSCs increased the autophagy level in IRI-challenged lungs. The protein levels of LC3, p62, and β-actin were examined using Western blotting analyses. (A) Representative Western blotting images of LC3 and p62 in IRI-induced lungs with or without BM-MSC pre-conditioning. (B and C) Statistical analysis of the expression of LC3-II/LC3-I and p62/β-actin in lungs. The results are represented as the mean ± SD. *P < 0.05 *versus* control group; ^#^*P* < 0.05 *versus* IRI group by anova (Bonferroni).

**Figure 4:**
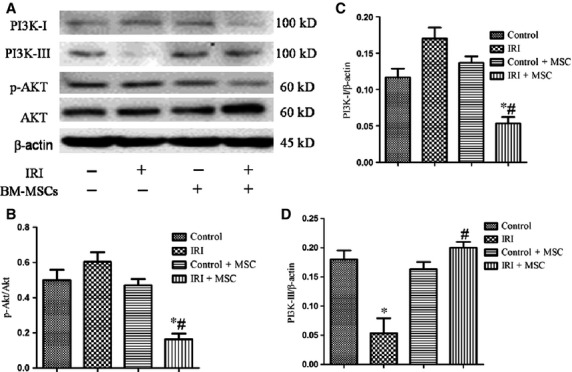
The role of the PI3K/Akt signalling pathway in mediating autophagy was defined using Western blotting analyses. (A) Representative Western blot image of PI3K class I, PI3K class III, and p-Akt in IRI-induced lungs with or without BM-MSC pre-conditioning. (B–D) Statistical analysis of the expression of p-Akt/Akt, PI3K class I/β-actin, and PI3K class III/β-actin in lungs. The results are represented as the mean ± SD. *P < 0.05 *versus* control group; ^#^*P* < 0.05 *versus* IRI group using anova (Bonferroni).

To elucidate the effect of BM-MSCs on autophagy in IRI lungs, we examined the expression of autophagic signalling markers using Western blotting analyses. Remarkably, we found increased expression levels of microtubule-associated protein LC3-II/LC3-1 and decreased level of p62 in the BM-MSCs treatment group compared to the IRI group (Fig. 3). Furthermore, the expression levels of PI3K class I and p-Akt were lower in the IRI+BM-MSCs group compared to the IRI group. In contrast, PI3K class III was increased in the BM-MSCs pre-treatment group compared to the IRI group (Fig. 4). Taken together, these data showed that transplantation of BM-MSCs promotes the autophagic mechanism *via* an up-regulation of autophagy-inducing factors, including PI3K class III, and LC3-II, as well as a down-regulation of negative regulators (PI3K class I, p-Akt) of autophagy in IRI.

### Effects of *in vitro* co-culturing of OGD-treated HPMVECs with BM-MSCs on cell death and permeability

**Figure 5:**
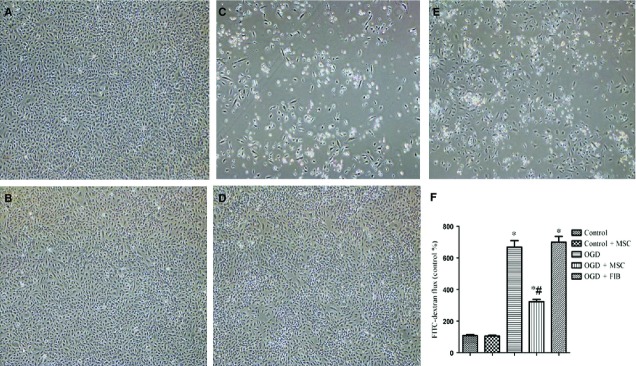
Protective effects of BM-MSCs on HPMVECs against oxygen-glucose deprivation (OGD). Phase contrast images of cultured HPMVECs with or without BM-MSCs: (A) Control group; (B) Control+BM-MSCs group; (C) OGD group; (D) OGD+ BM-MSCs group; (E) OGD+FIB group. (F) Administration of BM-MSCs alleviated OGD-induced high permeability in HPMVECs. This experiment was repeated three times in each group. Data are expressed as the mean ± SD. *P < 0.05 *versus* control group; ^#^*P* < 0.05 *versus* OGD group by anova (Bonferroni). FIB: fibroblast.

Morphological observations indicated marked alterations of HPMVECs after OGD treatment. We found that HPMVECs displayed less adhesion and more cell death compared to the normal condition. However, when the cells were co-cultured with BM-MSCs and not fibroblasts, significantly less cell damage and cell death were observed (Fig. 5A–E).

The permeability of HPMVECs was assessed by the passage of FITC-dextran through the endothelial monolayer. We found that OGD treatment significantly increased the permeability of HPMVECs compared to the normal condition (*P* < 0.05). Importantly, the permeability was significantly reduced when OGD-treated HPMVECs were co-cultured with BM-MSCs, but not with fibroblasts (Fig. 5F).

### Effects of *in vitro* co-culturing of OGD-treated HPMVECs with BM-MSCs on the mitochondrial membrane potential (ΔΨm)

**Figure 6:**
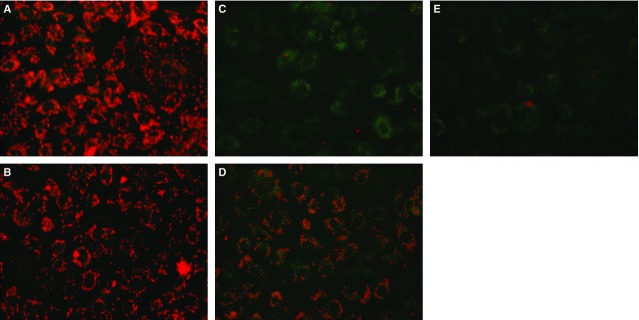
Effects of *in vitro* co-culture of OGD-treated HPMVECs with BM-MSCs on the mitochondrial membrane potential (ΔΨm). Red fluorescence indicates that JC-1 aggregates formed in cells with a high ΔΨm, whereas green fluorescence indicates that JC-1 monomers formed in cells with low ΔΨm. (A) Control group; (B) Control+BM-MSCs group; (C) OGD group; (D) OGD+ BM-MSCs group; (E) OGD+FIB group. FIB: fibroblast.

Human pulmonary micro-vascular endothelial cells were stained with JC-I dye and then observed using fluorescence microscopy. As shown in Figure 6, red fluorescence indicates that JC-1 aggregates formed in normal cells with a high ΔΨm, whereas green fluorescence indicates that JC-1 monomers formed in OGD-treated cells with a low ΔΨm. Importantly, ΔΨm was significantly increased when OGD-treated HPMVECs were co-cultured with BM-MSCs, but not with fibroblasts.

### Effects of an *in vitro* co-culture of OGD-treated HPMVECs with BM-MSCs on apoptosis and necrosis

**Figure 7:**
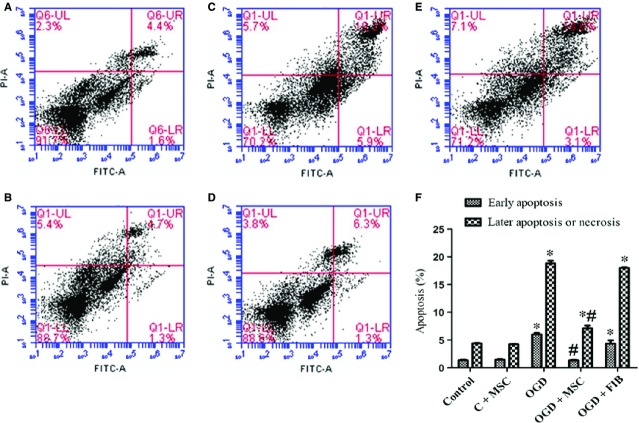
Effect of BM-MSCs on apoptosis and necrosis in OGD-induced HPMVECs. Cells were double-stained with Annexin V-FITC and PI and were then analysed using flow cytometry. This experiment was repeated independently three times in each group, and the representative results are shown. (A) Control group; (B) Control+BM-MSCs group; (C) OGD group; (D) OGD+BM-MSCs group; (E) OGD+FIB group. (F) Statistical analysis of the proportions of early apoptosis and later apoptosis of HPMVECs. These results are shown as the mean ± SD. *P < 0.05 *versus* control group; ^#^*P* < 0.05 *versus* OGD group by anova (Bonferroni). FIB: fibroblast.

The effects of BM-MSCs on apoptosis and necrosis of HPMVECs were analysed by FCM using Annexin V-FITC/PI dual staining. As shown in Figure 7, we observed a significantly higher proportion of early and late stage apoptotic HPMVECs cells in the OGD condition compared to the control condition (*P* < 0.05). The levels of both early and late stage apoptosis cells were markedly decreased when these OGD-treatment HPMVECs were co-cultured with BM-MSCs (*P* < 0.05). However, co-culturing of OGD-HPMVECs with fibroblasts did not affect the proportion of apoptosis (*P* > 0.05).

### *In vitro* co-culturing OGD-treated HPMVECs with BM-MSCs induces autophagy

**Figure 8:**
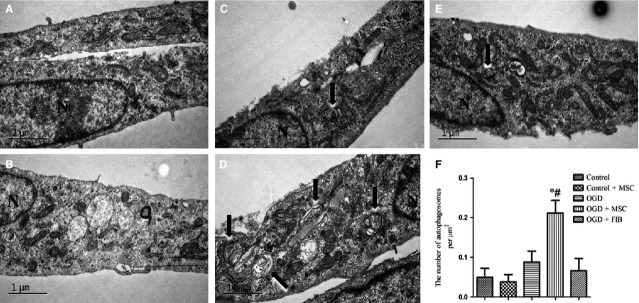
The autophagic levels in HPMVECs with or without BM-MSCs. Representative transmission electron microscopy (TEM) images for autophagy ultrastructures and quantitative analysis of the number of autophagosomes in different groups. (A) Control group; (B) Control+BM-MSCs group; (C) OGD group; (D) OGD+BM-MSCs group; (E) OGD+FIB group. (F) Statistical analysis of the number of autophagosomes per μm^2^. Autophagosomes are indicated by arrows. N = cell nucleus. The results are shown as the mean ± SD. *P < 0.05 *versus* control group; ^#^*P* < 0.05 *versus* OGD group by anova (Bonferroni). FIB: fibroblast.

**Figure 9:**
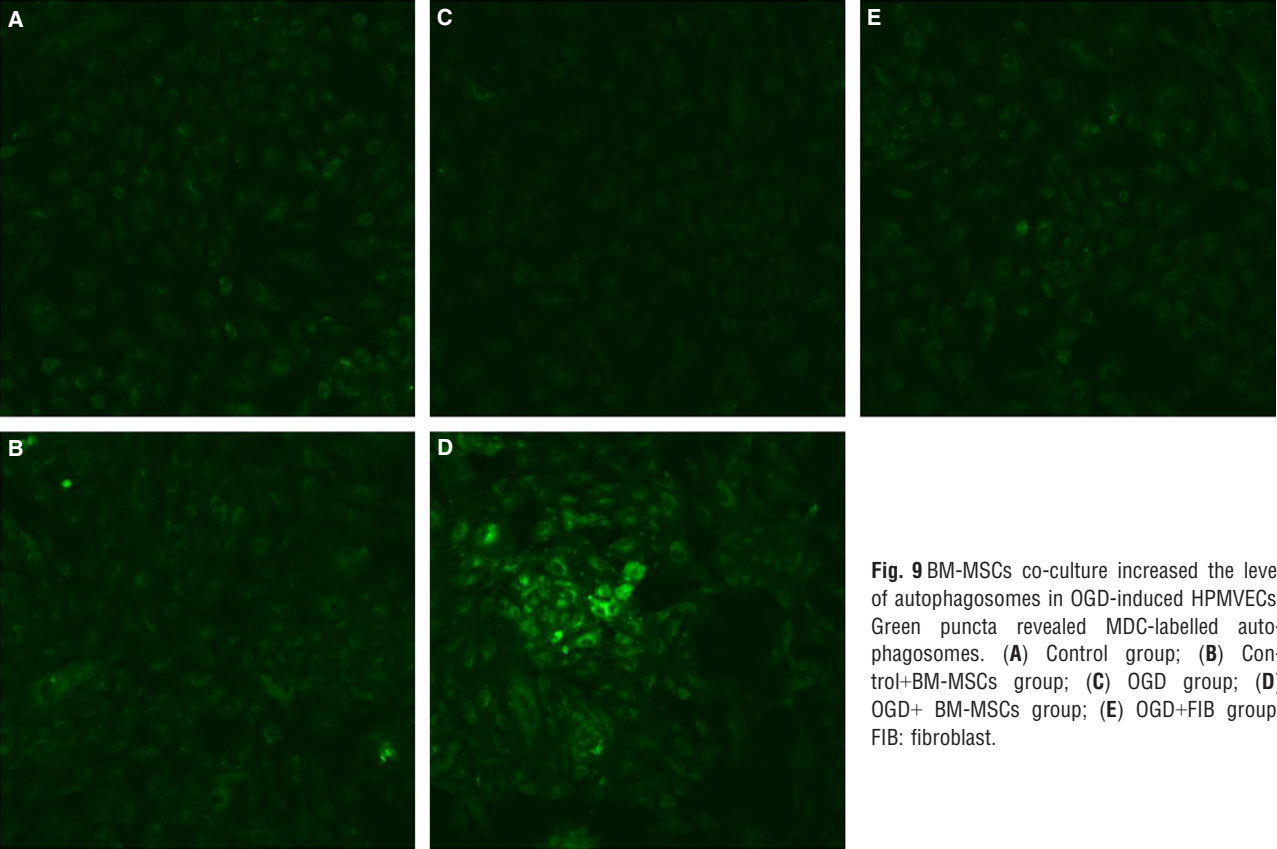
BM-MSCs co-culture increased the level of autophagosomes in OGD-induced HPMVECs. Green puncta revealed MDC-labelled autophagosomes. (A) Control group; (B) Control+BM-MSCs group; (C) OGD group; (D) OGD+ BM-MSCs group; (E) OGD+FIB group. FIB: fibroblast.

**Figure 10:**
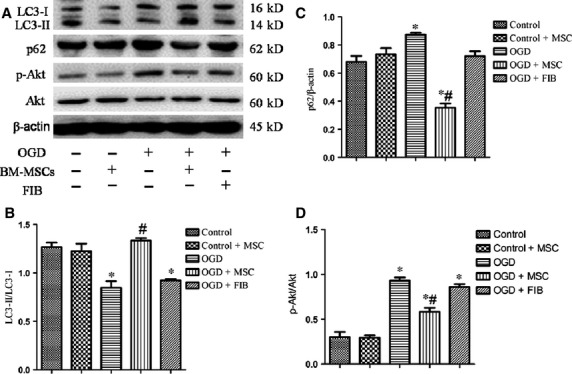
BM-MSC co-cultures increased the autophagy level in OGD-induced HPMVECs. The protein levels of LC3, p62, and β-actin were examined using Western blotting analyses. (A) Representative Western image of LC3-I, LC3-II, p62, and p-Akt in OGD-induced HPMVECs with or without BM-MSCs. (B–D) Statistical analysis of the expression of LC3-II/LC3-I, p62/β-actin, and p-Akt/Akt in OGD-induced HPMVECs. The results are shown as the mean ± SD. *P < 0.05 *versus* control group; ^#^*P* < 0.05 *versus* OGD group by anova (Bonferroni). FIB: fibroblast.

Next, we examined the effects of BM-MSCs on autophagy and their correlation with the PI3K/Akt signalling pathway in HPMVECs. We found that co-culturing HPMVECs with BM-MSCs, but not with control fibroblasts, induced autophagy under the OGD condition, as examined using TEM, MDC staining and Western blotting analyses. As shown in Figure 8, normal organelles and nuclei were observed in control cells, while many autophagosomes were detected in the OGD-induced HPMVECs pre-treated with BM-MSCs. Autophagosomes in HPMVECs were also detected using MDC staining. Green puncta revealed MDC-labelled autophagosomes and an increased number of autophagosomes as found in the OGD-treated HPMVECs co-cultured with BM-MSCs (Fig. 9). Furthermore, the ratio of LC3-II/LC3-I was elevated, while the expression of p62 was reduced in the BM-MSCs co-culture system compared to the OGD group. In addition, p-Akt/Akt was inactive during this process (Fig. 10).

To further investigate the role of PI3K/Akt pathway in autophagic activation, we treated HPMVECs with LY294002, a PI3K inhibitor, under the OGD condition. Interestingly, LY294002 treatment strongly diminished the ratio of LC3-II/LC3-I and increased the expression of p62 in OGD-treated HPMVECs co-cultured with BM-MSCs (Fig. 1).

**Figure 11:**
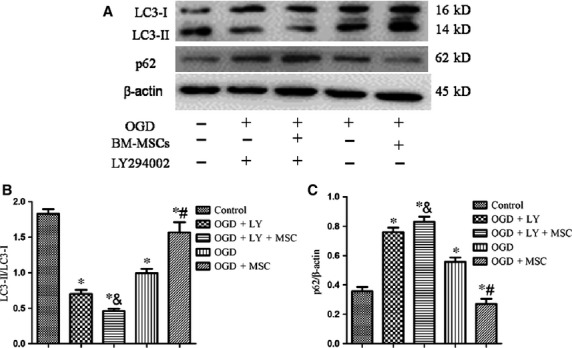
The role of PI3K/Akt in mediating autophagy was defined using Western blotting analyses. (A) Representative Western image of LC3-I, LC3-II and p62 in HPMVECs among the five groups. BM-MSC-induced autophagy was inhibited in response to LY294002. (B and C) Statistical analysis of the expression of LC3-II/LC3-I and p62/β-actin in OGD-induced HPMVECs. The results are shown as the mean ± SD. *P < 0.05 *versus* control group; ^#^*P* < 0.05 *versus* OGD group; ^&^*P* < 0.05 *versus* OGD+BM-MSCs group by anova (Bonferroni).

## Discussion

In this study, we confirmed that the transplantation of BM-MSCs in IRI-induced mice attenuated lung injury and exhibited anti-inflammation effects, as demonstrated by haematoxylin and eosin staining and a reduction in the inflammatory cytokine concentrations in BALF. Second, we found that the transplantation of BM-MSCs in IRI-induced mice triggered an induction of autophagy, as shown using Western blotting analyses with the autophagy markers, LC3-II/LC3-I and p62. In addition, *in vitro* co-cultures of HPMVECs with BM-MSCs, but not co-cultures with fibroblasts, similarly triggered an induction of LC3-II expression, strongly suggesting that BM-MSCs exert a specific autophagy-inducing effect. Third, our results demonstrated that PI3K/Akt signalling participated in the activation of autophagy by BM-MSCs. However, PI3K class I inhibited autophagy and PI3K class III stimulates autophagy.

Mesenchymal stem cells are multipotent cells that are derived from adult tissues that are capable of self-renewal and multi-directional differentiation. They are immune-privileged, as they express a very low level of major histocompatibility (MHC) class I and no MHC class II 18,19, which makes them a promising cell source for cellular therapy in lung disease 20,21. Recent studies have demonstrated that the administration of MSCs improved ALI, whether from *Escherichia coli* endotoxin or *E. coli* or following sepsis 22–24. In our study, we demonstrated that the transplantation of BM-MSCs could attenuate lung injury as well as reduce inflammatory cytokine levels induced by IRI. Furthermore, it also diminished the permeability of HPMVECs under the OGD condition. Although MSCs have shown promising therapeutic potential in lung injury and act *via* multiple signalling pathways and cellular events 25, the underlying mechanisms of their therapeutic effects remain poorly understood. Indeed, the balance between cell death and survival is critical for IRI. Thus, we examined whether BM-MSCs tip the balance between cell survival and apoptosis in lung injury. To investigate the regulation of different types of cell death, we evaluated the effects of BM-MSCs on apoptosis and necrosis. We observed that the OGD treatment of HPMVECs induced apoptosis and necrotic cell death. However, co-culturing HPMVECs and BM-MSCs under the OGD condition significantly reduced the proportion of apoptosis and necrosis. Taken together, these results strongly suggest that the beneficial effects induced by BM-MSCs involve a reduction of later apoptosis and necrotic death.

Autophagy, a form of ‘type II programmed cell death’, is closely related to cellular survival and homoeostasis under various stressors, such as starvation and hypoxic stress 5,26. Recently studies have shown that autophagy not only results in ‘cell death’ but also plays a role in ‘cell survival’ 27. However, despite the importance of autophagy in cell survival, apoptosis and necrosis, there has been no report on the regulation of autophagic mechanisms by BM-MSCs-related therapy in IRI. Thus, an important aspect of our study was to investigate whether the mechanism of autophagy is related to BM-MSCs-based therapeutic intervention. We found that the transplantation of BM-MSCs induced the activation of autophagy in an IRI-induced mouse model and OGD-induced HPMVECs model *via* the PI3K/Akt signalling pathway.

The PI3K/Akt signalling pathway plays a critical role in a wide range of cellular processes 10, including autophagy. It has been reported that PI3K class I and class III have opposing functions in the regulation of autophagy. Several studies have also shown that inhibiting the PI3K/Akt pathway suppressed LC3 levels 28. However, other studies have proposed that PI3K class I signalling inhibits autophagy because it activates the major downstream molecule of mammalian target of rapamycin (mTOR) 29, whereas PI3K class III signalling enhances autophagy by the induction of beclin 1 30. The roles of PI3K/Akt signalling in BM-MSCs transplantation-induced autophagy activation in an IRI-induced mouse model and OGD-induced HPMVECs model are still unknown. In this study, we provide several lines of evidence to support the view that PI3K class I/Akt signalling inhibits autophagy, while PI3K class III signalling enhances autophagy. Interestingly, when LY294002, a specific inhibitor of PI3K class I, was used in our study to investigate this mechanism, the autophagy induced by BM-MSCs under the OGD condition was reduced. One potential explanation may be that LY294002 has a greater effect on PI3K class I, but that autophagy is mostly regulated *via* PI3K class III.

To the best of our knowledge, this study is the first study to show that transplantation of BM-MSCs improves IRI *via* an up-regulation of autophagy-related signalling molecules. In addition, our results support the proposal that BM-MSCs stimulate autophagy in OGD-injured HPMVECs, at least in part *via* the PI3K/Akt signalling pathway. Moreover, there are some limitations in our study: First, we cannot confirm the identity of the cellular products or events that participate in BM-MSCs-induced autophagy. Second, the downstream molecular mechanism of PI3K/Akt in autophagy activation is unclear. Nevertheless, our findings show that transplantation of BM-MSCs involves the activation of autophagy and participation in the improvement mechanism, provide new insights into further understanding MSCs-based treatment and may provide novel avenues for the development of additional efficient therapies for diseases such as lung transplantation and cardiopulmonary bypass.

## Supporting Information

**Figure S1** Engraftment of BM-MSCs in lung tissues. Engrafted BM- MSCs stained with PKH26 (red) were detected three and a half hours after cell injection. The samples were stained using 6-diamino-2-phenylindole (DAPI) (blue).

**Data S1** Cell labelling.
