# Supplementary figures and images for: Bone marrow-derived mesenchymal stem cells enhance autophagy via PI3K/AKT signalling to reduce the severity of ischaemia/reperfusion-induced lung injury
Source: J Cell Mol Med. 2015 Jul 14;19(10):2341–51. doi: 10.1111/jcmm.12638 (PMC4594676; doi:10.1111/jcmm.12638)

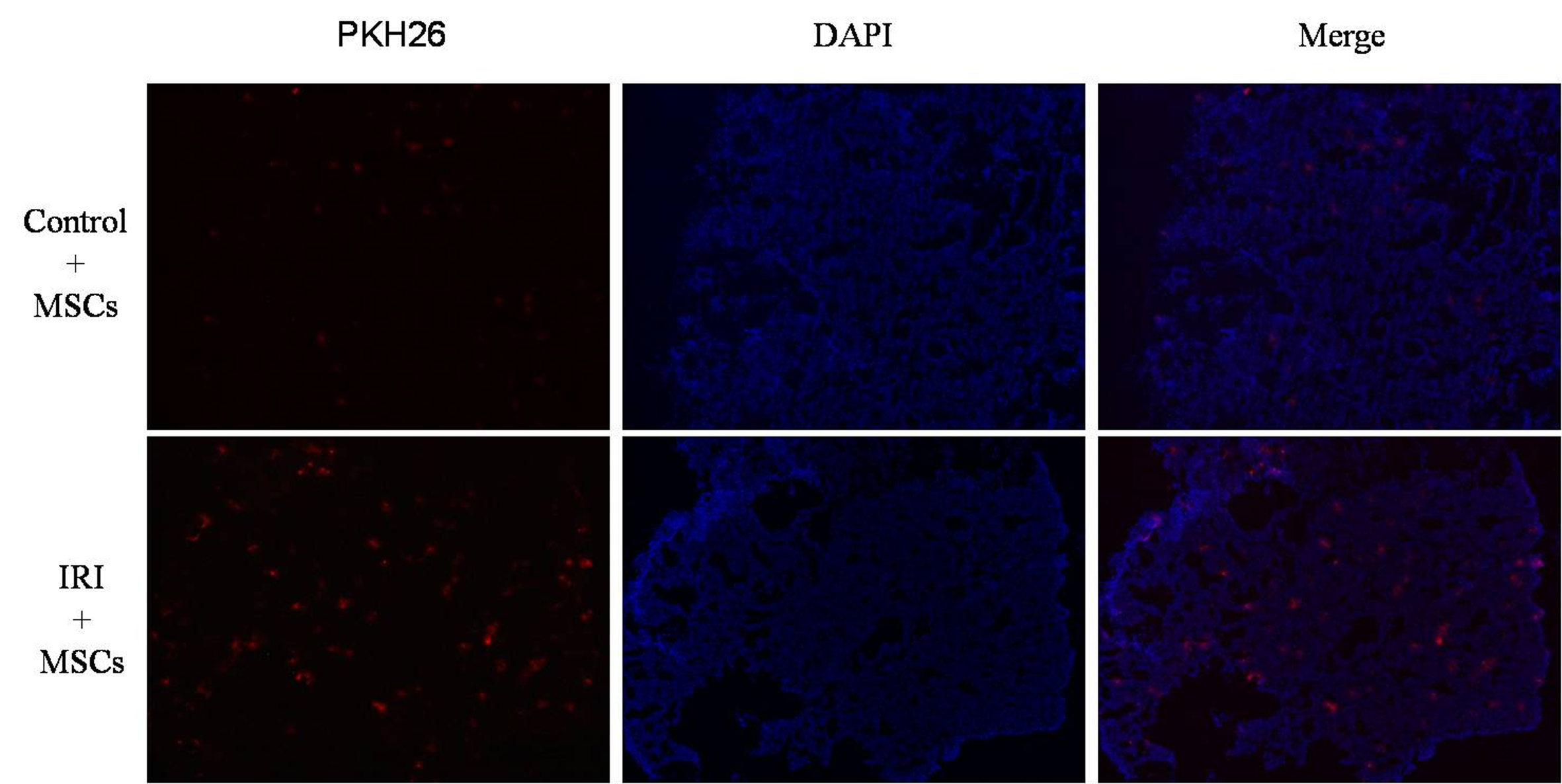

Supplement: Supplementary file 1 [file jcmm0019-2341-sd1.tif]
