# Supplementary material for: Bone marrow-derived mesenchymal stem cells enhance autophagy via PI3K/AKT signalling to reduce the severity of ischaemia/reperfusion-induced lung injury
Source: J Cell Mol Med. 2015 Jul 14;19(10):2341–51. doi: 10.1111/jcmm.12638 (PMC4594676; doi:10.1111/jcmm.12638)
Supplement: Supplementary file 2 [file jcmm0019-2341-sd2.pdf]

Bone marrow-derived mesenchymal stem cells enhance autophagy via PI3K/AKT signalling to reduce the severity of ischemia/reperfusion-induced lung injury

Jing Li, M.D.[1,2#]; Jian Zhou, M.D., Ph.D.[1,2#]; Dan Zhang, M.D.[1,2]; Yuanlin Song, M.D., Ph.D.[1,2]; Jun She, M.D., Ph.D.[1,2*]; Chunxue Bai, M.D., Ph.D., FCCP[1,2*]

Affiliation: 1 Department of Pulmonary Medicine, Zhongshan Hospital, Fudan University, Shanghai, China; 2 Shanghai Respiratory Research Institute, Shanghai, China.

[*]Please address correspondence to:

Chunxue Bai, M.D., Ph.D.

Department of Pulmonary Medicine, Zhongshan Hospital, Fudan University, Shanghai, China; Shanghai Respiratory Research Institute, Shanghai, China;

No.180, Fenglin Road, Xuhui District, Shanghai, 200032, People's Republic of China

Tel: +86-21-64041990-3077

E-mail: bai.chunxue@zs-hospital.sh.cn

Jun She, M.D., Ph.D.

Department of Pulmonary Medicine, Zhongshan Hospital, Fudan University, Shanghai, China; Shanghai Respiratory Research Institute, Shanghai, China;

No.180, Fenglin Road, Xuhui District, Shanghai, 200032, People's Republic of China

E-mail: shejuncn@aliyun.com

[#] These authors share co-first authorship.

Running Title: Bone marrow-derived mesenchymal stem cells enhance autophagy to reduce the severity of acute lung injury

Supplementary Materials and Methods

Cell labelling

BM-MSCs were labelled using a PKH26 Fluorescent Cell Linker Kit (Sigma) according to the manufacturer's instructions, resuspended in PBS at a density of 5×10^6 cells/ ml and then kept on ice prior to transplantation.

Supplementary figure legends

Figure 1.

Engraftment of BM-MSCs in lung tissues.Engrafted BM-MSCs stained withPKH26 (red) were detected three and a half hours after cell injection. The samples were stained using 6-diamino-2-phenylindole (DAPI) (blue).
